# Data integration to prioritize drugs using genomics and curated data

**DOI:** 10.1186/s13040-016-0097-1

**Published:** 2016-05-26

**Authors:** Riku Louhimo, Marko Laakso, Denis Belitskin, Juha Klefström, Rainer Lehtonen, Sampsa Hautaniemi

**Affiliations:** Genome Scale Biology Research Program, Research Programs Unit, Faculty of Medicine, University of Helsinki, P.O. Box 63 (Haartmaninkatu 8), Helsinki, FI-00014 Finland; Translational Cancer Biology Research Program, Research Programs Unit, Faculty of Medicine, University of Helsinki, P.O. Box 63 (Haartmaninkatu 8), Helsinki, FI-00014 Finland

**Keywords:** Data integration, Drug prioritization, Gene ontology, Cancer, Breast cancer

## Abstract

**Background:**

Genomic alterations affecting drug target proteins occur in several tumor types and are prime candidates for patient-specific tailored treatments. Increasingly, patients likely to benefit from targeted cancer therapy are selected based on molecular alterations. The selection of a precision therapy benefiting most patients is challenging but can be enhanced with integration of multiple types of molecular data. Data integration approaches for drug prioritization have successfully integrated diverse molecular data but do not take full advantage of existing data and literature.

**Results:**

We have built a knowledge-base which connects data from public databases with molecular results from over 2200 tumors, signaling pathways and drug-target databases. Moreover, we have developed a data mining algorithm to effectively utilize this heterogeneous knowledge-base. Our algorithm is designed to facilitate retargeting of existing drugs by stratifying samples and prioritizing drug targets. We analyzed 797 primary tumors from The Cancer Genome Atlas breast and ovarian cancer cohorts using our framework. FGFR, CDK and HER2 inhibitors were prioritized in breast and ovarian data sets. Estrogen receptor positive breast tumors showed potential sensitivity to targeted inhibitors of FGFR due to activation of FGFR3.

**Conclusions:**

Our results suggest that computational sample stratification selects potentially sensitive samples for targeted therapies and can aid in precision medicine drug repositioning. Source code is available from http://csblcanges.fimm.fi/GOPredict/.

**Electronic supplementary material:**

The online version of this article (doi:10.1186/s13040-016-0097-1) contains supplementary material, which is available to authorized users.

## Background

Finding the right drug for the right patient is an integral part of precision medicine and computational methods to facilitate matching patients to drugs are urgently needed [1]. Patient stratification using clinical or molecular features to identify patients that most likely respond to a drug allows reducing costs in drug development [2], maximizing the number of responding patients [3], and minimizing side-effects to non-responding patients [4]. Patient-stratified analysis in a cancer may result in suggestions of drugs that have not been indicated in cancer care earlier. This so called drug repositioning offers novel opportunities to find effective treatments for cancer patients.

The molecular landscape of a tumor affects the efficacy of several drugs and is central for clinical trial design for targeted therapies [5]. In particular, molecular level alterations, such as point mutations, somatic copy-number amplifications and promoter hypomethylation, play key roles in both stratifying patients and finding drugs for repositioning [6]. For instance, genomic alterations affecting the production of drug target proteins occur in several tumor histological types as exemplified by druggable HER2 mutations in both breast and metastatic gastric cancer [3]. These drug target proteins, which are genomically altered in multiple cancers, are thus prime candidates for precision medicine drug repositioning [7, 8]. In addition, utilization of signaling networks offers possibilities for improving cancer drug treatments [9].

The large variety of molecular level alterations in cancers calls for computational data integration methods to enable precision medicine via improved patient stratification and drug repositioning [10–12]. Most integration methods use two or three types of molecular alterations and seldom incorporate in a single algorithm signaling pathway or curated information available in databases [13, 14]. For instance, [15] used transcriptomics data in drug prioritization whereas the MOCA algorithm integrated genomics data with Boolean set operations to build multigene-modules to predict drug responses and stratify cell lines in a pan-cancer setting [16]. In particular, knowledge available in open-access cancer genomic studies represents a large untapped resource for enhancing interpretation of analysis results.

We introduce here a computational algorithm called GOPredict that allows patient stratification and drug repositioning via comprehensive integration of genomics data, signaling pathway information, drug target databases and curated knowledge in databases. We demonstrate the utility of GOPredict by stratifying Cancer Genome Atlas (TCGA) breast and ovarian cancer samples and prioritizing drugs in these two cohorts [17–19].

## Methods

**Fig. 1:**
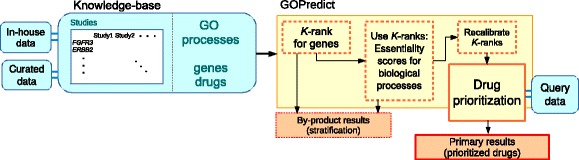
Conceptual overview of GOPredict. In-house and curated data (*left*) are used to create a gene-by-study matrix of ranks which is stored in the knowledge-base (*large blue box*). GOPredict uses the genewise study ranks to calculate gene *K*-ranks (*yellow box*, *left*). *K*-ranks are used to calculate cancer-essentiality for GO processes (*yellow box*, *middle*). *K*-ranks are recalibrated with GO process scores and then used to prioritize drugs and stratify samples for input query data sets

Our data integration approach consists of two major steps. First, we have developed a knowledge-base that contains molecular, drug information and analysis results from multiple public databases and private sources. Second, we have developed an algorithm (GOPredict) to mine the knowledge-base. An overall schematic of the approach is given in Fig. 1.

The major design principles of the knowledge-base are as follows. First, the knowledge-base is gene-centric (meaning that the information in the knowledge-base is associated to a gene identifier) because this allows taking into account published results that are mostly gene-centric. This design also allows automated analysis of drug-gene target-pairs. Second, results in the knowledge-base are stored as ranks. The use of ranks enables comparing and combining data over multiple data sets, data types, and measurement technologies as well as between numerical, ordinal and categorical data [20]. In addition, rank-based scoring is less biased towards well-studied genes [21].

GOPredict uses signaling pathway information defined with Gene Ontology (GO) biological processes [22]. The Gene Ontology contains high level processes (e.g., ’apoptosis’) as well as specific signaling pathways (e.g., ’ERBB signaling pathway’). Unlike other signaling pathway databases [23], the GO and its standardized naming conventions for biological processes provide a flexible and reliable data source to define signaling pathway context for genes [24].

The underlying modeling question for GOPredict is “what is the best drug to target proteins affected by genomic aberrations and driving tumorigenic signaling in tumors”. The GOPredict algorithm is described in detail in Additional file 1. Briefly, gene-based rank data from the knowledge-base is related to the GO processes that genes regulate. Each gene-drug target pair is then prioritized based on the GO processes, that the gene regulates, and the priority rank of a drug is averaged over all genes which the drug regulates. In this section we use select examples to provide a general description of the knowledge-base and GOPredict.

### Data sets

We gathered results of genomic, transcriptomic, and epigenomic (DNA methylation), and descriptive (gene-phenotype connections) analysis data from nine public cancer data sets. Each data set consisted of one or more of these data levels. There are two sources of data which we call **in-house data** and **curated data**. In-house data comprise raw data that we downloaded and analyzed. Curated data comprise analyzed gene-level result data that we downloaded from the source databases and did not process further. A short description of studies is in Table 1 and a more detailed list in Additional file 2.

**Table 1:** List of in-house (TCGA) and curated data sets in the knowledge-base. A more detailed description of each data set, data type and study is in Additional files 1 and 2

Data source	Study type	Number of studies
In-house (analysis based)	Somatic CNA (gain frequency, deletion frequency, survival)	11
	DNA methylation (survival)	4
	Expression (survival, fold-change)	8
Curated (literature based)	Amplified and overexpressed cancer genes	1
	Breast cancer brain metastasis genes	1
	Cancer Gene Census activated	1
	Cancer Gene Census inactivated	1
	COSMIC	3
	Tumorscape	20

The in-house data comprise four Cancer Genome Atlas (TCGA) primary tumor data sets totaling approximately 2,200 samples of breast, ovarian, colorectal and glioblastoma brain cancer [17–19, 25]. Three of the data sets, glioblastoma, breast and ovarian cancer, we had previously analyzed [26–28]. The curated data comprise Tumorscape [29], COSMIC [30, 31], the Cancer Gene Census genes [30], the amplified and overexpressed genes in cancer collection [32], and a breast cancer brain metastasis gene collection [33] (Additional file 1). The download and analysis of the in-house data are automated using Anduril computational infrastructure [26]. Details of the in-house analysis of the TCGA data are provided in Additional file 1.

### Knowledge-base

The knowledge-base comprises analysis results from the in-house and curated data sets. Conceptually, in-house and curated data are composed of one or more **studies** in the knowledge-base. A study is a ranked list of genes that are ranked based on a statistical analysis of a molecular data type in a specific cancer or literature source. All studies are stored gene-wise and for each gene the database contains its rank order in each study (Methods, Additional file 1). For each study only those genes, which meet study specific inclusion criteria, receive ranks and are connected to a study. For example, a gene is ranked based on its fold-change in an in-house data expression analysis if the difference in means of gene expression values between tumor and control samples is significant (t-test *q*≤0.001, Benjamini-Yakutieli multiple hypothesis correction [34]). Full details of all inclusion criteria are given in Additional file 1.

Studies can be combined into study sets. Users can tailor and modify study sets flexibly to suit different research questions. We provide three default study sets: **activating** (containing e.g., gene upregulation and gene copy-number increase results from the four in-house TCGA data sets), **inactivating** (e.g., gene downregulation, gene copy-number deletion), or **survival-associated** (univariate association of gene copy-number increase with overall survival). A gene may belong to one or several studies and study sets. A full list of studies in each default study set is in Additional file 1. The default study sets were constructed conservatively only to contain studies which unambiguously fit into these study set definitions.

In addition to gene ranks in studies, the knowledge-base contains drug gene-target information from KEGGDrug [35] and DrugBank [36], and signaling pathways from the Gene Ontology (http://geneontology.org/, downloaded August 2013). This compound design enables rapid integration, combination and comparison of data over multiple data sets, data types, and measurement technologies. KEGGDrug, DrugBank and Gene Ontology are stored in the database as described in Additional file 1 and by [37]. DrugBank and KEGGDrug contain approved drugs and experimental compounds. For notational convenience, we use the word ‘drug’ to refer to all compounds retrieved from DrugBank and KEGGDrug.

### Cancer-essentiality scoring and gene ranks

The overall goals of the GOPredict algorithm are to prioritize drugs with known protein targets, characterize genes, and stratify samples. GOPredict works in four steps (Additional file 1: Figure S1). The first and second steps are preparatory. In the third step samples are stratified and in the fourth drugs are prioritized.

First, gene ranks are extracted from the database for each study and used to calculate normalized gene ranks called the *K*-ranks (Additional file 1: Figure S1a). For example, the fibroblast growth factor receptor 3 (*FGFR3*) has a rank in two studies which are used to calculate its *K*-rank. The two studies for *FGFR3* are a curated study (*activating mutations in the Cancer Gene Census*) and an in-house study (*differential gene expression in TCGA breast cancer*). Each *K*-rank quantifies the cancer-essentiality of a gene.

Second, since genes are connected to GO processes, the *K*-ranks are used to calculate GOPredict cancer-essentiality scores for GO processes (Additional file 1: Figure S1b). The higher the score, the more cancer-essential the GO process is. For example, 1130 genes negatively and 939 positively regulate ‘cell development’ (GO:0048468) and have a *K*-rank in the database (see previous section and Additional file 1 for *K*-rank inclusion criteria). The *K*-ranks of these genes from step one are summed up to produce the cancer-essentiality score for ‘cell development’ and statistical significance is assessed with a permutation test (Additional file 1).

### Sample stratification and drug prioritization

Before explaining the third and fourth steps of GOPredict, we first need to clarify inputs to GOPredict. The input to the third and fourth steps of GOPredict is called **a query data set** (Fig. 1). A query data set consists of molecular data for a set of samples in which we want to stratify samples for drug prioritization. The third and fourth steps of GOPredict produce the drug prioritization and sample stratification for an input query data set.

To prioritize drugs, we first construct for each query data set an **activity matrix** using a set of biologically motivated logical rules based on the molecular measurement data such gene expression, gene copy-number and mutation data (Additional file 1). The activity matrix is a gene-by-sample binary matrix denoting the status (active, inactive, unchanged) of a gene. The status of a gene is preferably defined by its expression status. In cases where a gene’s expression status conflicts with its copy-number state and copy-number is altered, copy-number takes precedence (full details in Additional file 1). The rationale for prioritizing genomic alterations is that they are more stable and reproducible over different studies than expression level alterations, and therefore are more viable as candidate biomarkers [38]. The activity matrix is also used when interpreting results of drug prioritization because sample stratification is extracted from the activity matrix.

In step three, the GO processes’ cancer-essentiality score *P*-values are used to recalibrate gene *K*-ranks. The recalibrated *K*-rank is the harmonic mean of *P*-values of all GO processes a gene regulates (Additional file 1: Figure S1c). In ambiguous cases where a gene is annotated both as a positive and negative regulator of a GO process, that GO process is not used in the calculation. For example, *FGFR3* unambiguously regulates 17 GO processes, 9 positively and 8 negatively, of which two are depicted in Additional file 1: Figure S1c. The recalibration 1) connects signaling pathways to drug target genes and 2) normalizes the scores so that highly connected processes (terms that are high in the GO hierarchy and therefore connected to more genes) do not dominate the results. Without recalibration, drug scores would be biased towards more highly connected biological processes. Only a subset of genes receive recalibrated ranks. Genes that code for drug target proteins in the knowledge-base and are in the activity matrix (implying they are altered in the query data set) are used for prioritization. Other genes are removed and the final set of genes only contains genes that are drug targets.

In step four, recalibrated gene *K*-ranks are used to prioritize drugs (Additional file 1: Figure S1d). The prioritization score balances (1) the number of targets of a drug; (2) the relevance of the drug targets according to the database; and (3) the measured activity of the target gene in input cancer data.

## Results

To demonstrate the use of the knowledge-base and GOPredict, we downloaded and analyzed with GOPredict 497 primary breast carcinoma (BRCA) and 390 ovarian adenocarcinoma (OVCA) tumors from the Cancer Genome Atlas [18, 19]. We constructed activity matrices for each cancer by fusing mutation, copy-number, and expression data. All reported *P*-values are nominal as they are only used for ranking.

### Cancer-essentiality prioritizes known cancer genes

A byproduct of the knowledge-base design is that it allows defining hypothesis-driven selection of study sets and calculating cancer-essentiality in the study sets. A full list of studies in each study set is in Additional file 2. Study sets can be flexibly redefined by the user and the knowledge-base is user-extendable with additional studies.

We used GOPredict to characterize the cancer-essentiality of genes in activating, inactivating and survival-associated study sets using the *K*-rank. The genes, which GOPredict characterized to be cancer-essential, include known cancer genes such as *EGFR*, *ERBB2* and *FGFR3*, tumor suppressors such as *RB1*, *TP53* and *PTEN* as well as genes not previously associated with cancer (full results in Additional files 1, 3 and 4). This analysis shows that the *K*-rank accurately prioritizes cancer genes based on data in the knowledge-base.

### Kinase inhibitors are prioritized in primary breast tumors

In addition to cancer-essentiality, we prioritized drugs in two primary tumor data sets with GOPredict. The drug priorization analysis contains only those drugs that have at least one altered gene target in either BRCA or OVCA activity matrices. Out of 1559 drug-gene pairs in the knowledge-base, we calculated GOPredict scores for a total of 504 drugs in BRCA and 493 drugs in OVCA. Of the drugs 269 overlapped between the two cancer types.

As a proof-of-concept, we first analyzed a query data set containing all BRCA samples with a immunohistochemically verified *ERBB2* amplification according to TCGA clinical data. In breast cancer, *ERBB2* amplification is an established indicator to use *ERBB2* inhibitors with notable success [39]. As expected, drugs targeting *ERBB2* dominated the results with four *ERBB2* inhibitors among the 10 best scoring drugs (Additional file 4). This analysis shows that GOPredict accurately prioritizes subtype-specific drug targets when such exist. Thus, for a novel cancer subtype defined with molecular features, GOPredict could immediately suggest efficient interventions.

To test the sensitivity of GOPredict to the choice of study sets, we added three TCGA methylation studies and re-analyzed the *ERBB2* amplified query data set. In addition, we performed a second re-analysis on the same data where instead of adding we removed two studies. Results from both re-analyses were highly concordant with the original analysis for both cancer-essentiality and drug prioritization scores (Additional file 1). This suggests that GOPredict scoring is robust to changes in study sets.

**Fig. 2:**
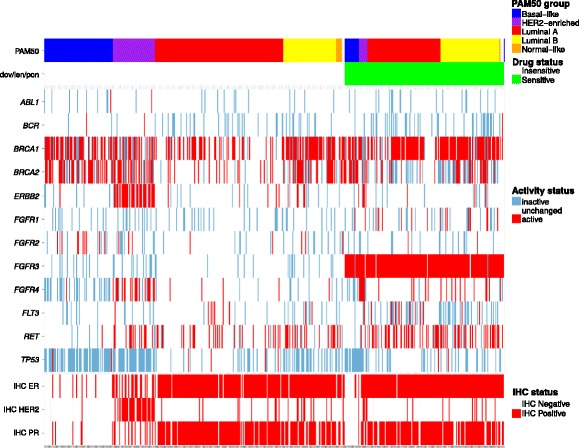
Heat map of sample stratification according to *FGFR3* status in TCGA breast tumors. Breast cancer tumors are on the x-axis. Y-axis contains gene activity matrix statuses and immunohistochemical (IHC) status of ER, PR and HER2. PAM50 subtype classification is on the top-most row. FGFR inhibitors dovitinib, lenvatinib and ponatinib (dov/len/pon) share sensitive samples (*green*). Samples have been ordered according to FGFR inhibitor sensitivity status

To obtain a general view on drug sensitivity patterns in breast cancer, we analyzed the entire BRCA cohort. Drugs targeting matrix metalloproteinases and fibroblast growth factor receptors (FGFR) are ranked the highest in the entire sample set (Additional file 4). FGFR inhibitors have the largest patient group for therapeutic targeting (174–211 sensitive samples, 35–42 % of samples, Fig. 2). Drugs targeting the Smoothened protein (erismodegib, saridegib and vismodegib) are also among the ten highest ranking drugs (34 samples).

### Sample stratification shows luminal breast cancers as putative targets of FGFR inhibition

Sample stratification according to sensitivity to FGFR inhibitors dovitinib, lenvatinib and ponatinib is shown in Fig. 2. The figure depicts a categorical heat map containing activity matrix statuses of target genes that were used in the sensitivity prediction (*ABL1, BCR, FTL3* and *RET*), all *FGFR* family members (*FGFR1-4*) and possible confounders (*BRCA1, BRCA2, TP53* and *ERBB2*). In addition, the immunohistochemical staining status of estrogen receptor, progesterone receptor and HER2 receptor are shown. Samples sensitive to the three drugs were assigned almost exclusively according to *FGFR3* activation status (97 % overlap, Fig. 2). The sensitive samples for all three drugs overlapped completely.

To further characterize the sensitive samples, we compared GOPredict’s strata to the PAM50 subtypes. PAM50 is a gene expression based molecular subtyping method for breast cancer and is well established [40]. FGFR inhibitor sensitive samples comprised samples from every PAM50 breast cancer molecular subtype but exhibited a clear enrichment of luminal samples. Basal, HER2-enriched and normal samples showed no differences in the proportion of sensitive samples (Fisher’s exact test *P*=1). The proportion of sensitive samples in these three subtypes differed significantly from luminal A (Fisher’s exact test *P*=0.0006) and luminal B proportions (Fisher’s exact test *P*=0.00001). In addition, FGFR3 inhibitor sensitive samples were enriched in luminal B samples when compared directly with luminal A (Fisher’s exact test *P*=0.004). In summary, luminal subtypes in general and preferentially luminal B breast cancer samples were significantly enriched for FGFR inhibitor sensitive samples according to activity patterns of FGFR inhibitor targets.

### GOPredict prioritizes kinase inhibitors in an independent ovarian cancer cohort

In addition to breast cancer, we tested GOPredict in an independent set of ovarian adenocarcinoma primary tumors. In OVCA (Additional file 4), CDK inhibitors (dinaciclib, alsterpaullone) received substantially higher ranks (first and second) and a large number of sensitive samples (308 to 356 samples). The multi-target tyrosine kinase inhibitor bosutinib attained the third highest score and a comparatively large number of sensitive samples (341 samples). All in all, the top ten scoring drugs in ovarian sample set were enriched for CDK specific inhibitors (7/10 drugs).

## Discussion

In precision medicine, molecular markers are used to tailor drug treatment for patients to maximize clinical benefit [8]. The large number of available compounds has led to a need to match molecular profiles of a tumor to a potentially effective therapy. Accordingly, integrative computational methods are needed to match patient strata to appropriate drugs.

We have presented here a novel approach to facilitate precision medicine via the use of pathway and existing public data as well as an integrative framework that fuses multiple types of molecular data from tumors. GOPredict is based on a knowledge discovery concept that allows “data to speak”. As shown by the knowledge-based Gene Set Enrichment Analysis framework [41], statistical testing may be too restrictive and sometimes impossible to apply to multi-dimensional data sets since it is hard to establish null and alternative hypotheses. The knowledge-base’s modular and extensible design allows defining study sets flexibly for new research questions. Furthermore, the rank-based scoring design in GOPredict enables the integration and comparison over varied types of cancer, measurement technology and data scales.

GOPredict prioritized FGFR inhibitors as the major class of putatively effective therapeutics in breast cancer. Signaling via FGFR family members plays a role in tumorigenesis and drug sensitivity in breast cancer [42] and other solid tumors [43]. Our results suggest the involvement of FGFRs in breast and ovarian cancer and that a substantial proportion of breast tumors are potentially sensitive to FGFR inhibition. Pan-kinase inhibitors have varied binding affinity to their target proteins [44] but these data are not feasibly available for automated algorithms.

Five of the top ten prioritized drugs for breast cancer were FGFR inhibitors. All five are in Phase 2 or 3 trials for multiple cancers [45] and pazopanib as well as dovitinib have active breast cancer trials (https://clinicaltrials.gov/, Accessed 25 Jan 2015). Many of our predicted sensitive samples harbored genomic alterations in FGFRs. One of the first clinical breast cancer studies where the selection of patients was based on *FGFR1* amplification status, found dovitinib to reduce tumor size more in *FGFR1* amplified than non-amplified patients [46].

The samples predicted to be FGFR inhibitor sensitive were almost exclusively *FGFR3* activated and were enriched for PAM50 luminal A and B breast cancer subtypes. Luminal breast cancers are characterized by estrogen receptor (ER) positivity [40]. Tamoxifen is a targeted estrogen receptor inhibitor used for adjuvant endocrine treatment of estrogen or progesterone receptor positive breast tumors [47]. Interestingly, FGFR3 expression is higher in breast tumors that are resistant to tamoxifen [48] and high expression of *FGFR4* predicts poor response to tamoxifen therapy in primary tumors [49]. Furthermore, invasive lobular breast carcinoma cell lines are sensitive to a combined inhibition of ER and FGFR activity [50]. Our results suggest that this sensitivity to combinatorial treatment is due to activation of FGFR3.

Tamoxifen resistant breast tumors have been found to be sensitive to vismodegib (Smoothened antagonist) in xenograft mice [51]. In our analysis vismodegib was one of the three Smoothened inhibitors in our priority list in breast cancer. This suggests that tamoxifen resistant breast tumors could benefit from a combinatorial therapy with Smoothened and FGFR inhibitors.

In our breast cancer data, roughly 20 % of HER2 positive tumors had potentially activating alterations in *FGFR3*. According to current guidelines, HER2 positive patients are pharmacologically treated with HER2 inhibitors [52]. Our results with GOPredict suggest that HER2 inhibitor insensitive tumors could be amenable to treatment with FGFR3 inhibitors and that a fifth of patients would stand to benefit from this treatment.

Amiloride was the highest ranked drug in HER2 positive tumors. Amiloride is a pyrazine compound used to treat hypertension and heart failure. Interestingly, amiloride and its derivatives have been recently suggested to have anti-cancer effects in breast cancer cells independent of subtype [53, 54]. An earlier study, however, found amiloride to increase cell motility in HER2 positive breast cancer cells [55]. Taken together, these results warrant further study to determine the applicability of amiloride to breast cancer.

In ovarian cancer, seven out of ten top priority drugs are cyclin-dependent kinase (CDK) inhibitors. CDKs are a family of protein kinases that participate in the cell cycle and are targeted by several inhibitors [56]. Several CDKs are potential oncogenes including *CDK4* in ovarian cancer [57]. To date, several CDK inhibitors are in Phase 2 and 3 trials [58] and of the seven CDK inhibitors in our result list flavopiridol is undergoing Phase 2 trials in ovarian cancer as a combination treatment with oxaliplatin or cisplatin (https://clinicaltrials.gov/, Accessed 25 Jan 2015). Moreover, dinaciclib, the highest scoring CDK inhibitor by GOPredict, has been shown to sensitize ovarian cancer cell lines to platinum drugs via downregulation of BRCA1 [59, 60]. These results suggest that a sizable fraction of ovarian tumors are potentially sensitive to CDK inhibitors when combined with chemotherapy.

Our knowledge-base contains both in-house and curated microarray data sets from multiple microarray platforms and sources. Since GOPredict is designed to be extendable and to contain data sets from multiple sources, preprocessing steps such as normalization cannot be fully standardized and can therefore induce some noise. Nonetheless, our results with varying study sets indicate that GOPredict scoring is robust to noise in the study data.

GOPredict is dependent on centralized and frequently maintained databases such as GO and Ensembl. Many databases, however, undergo changes in both data content and database interfaces. These changes increase the maintenance burden of tools such as GOPredict. GOPredict could be improved in the future in three ways: (1) the addition of binding-affinity of drug-target pairs to weight each drug target gene; (2) inclusion of data on drug combinations and synthetic lethal interactions and; (3) addition of more result databases. Data on the first two points are currently scattered and automated retrieval is infeasible. GOPredict utilizes many result databases but this list is incomplete. For example, the Comparative Toxicogenomics Database (CTD) contains special disease related GO annotations as well as breast and ovarian cancer marker gene studies which could be incorporated into GOPredict [61].

GOPredict results can in future work guide experimental design. For example, top scoring drugs from our GOPredict analysis could be administered to breast cells with suitable genetic profiles to test their efficacy in vitro.

GOPredict produces several by-product results when prioritizing drugs. For example, *SLC25A32* received high cancer-essentiality scores through alterations in both ovarian and breast cancer study sets which could indicate a role for *SLC25A32* in these cancers. Accordingly, we built a multivariate Cox survival model in TCGA OVCA data and found that the overexpression of *SLC25A32* (ANOVA *P*=0.003) and lack of residual tumor (ANOVA *P*=0.02) were significant independent predictors of poor survival (Additional file 1). SLC25A32 is folate transporter localized in mitochondria [62]. Folates are required for DNA replication in cell division and have a dual role in cancer drug efficacy [63]. Since ovarian tumors express moderate levels of SLC25A32 [64], our results suggest that ovarian tumors might be sensitive to antifolate chemotherapy substances such as methotrexate which is pending results from clinical trials (https://clinicaltrials.gov/, Accessed 25 Jan 2015).

## Conclusions

Here we present GOPredict, which is a novel approach to integrate information from multiple public sources, signaling pathway and drug target data with local genomic data in cancer. Our results suggest that GOPredict can augment current pathology-based definitions of patient groups for targeted drug therapies which can potentially benefit many cancer patients. A practical application for GOPredict is to screen genomic measurements of cancer model systems for previously overlooked druggable genomic alterations and simultaneously prioritize which drugs to screen.

Our approach is able to infer kinase inhibitors as highly relevant drugs in breast and ovarian cancer based solely on signaling pathway information, pre-existing genomic result data and molecular measurement data. These inhibitors are prime candidates for further testing in drug repositioning experiments. Furthermore, our results highlight none-cancer drugs such as amiloride which have only recently been tested for anti-cancer efficacy with promising results. Our primary results indicate that FGFR inhibitors in breast cancer and CDK inhibitors in ovarian cancer as well as pazopanib in both cancers are predicted to have the largest proportion of putatively sensitive samples.

## Additional files

Additional file 1 A PDF-document containing the Supplementary Figures, Results and Methods. (PDF 273 kb)

Additional file 2 A spreadsheet file containing the list of studies. (XLS 15 kb)

Additional file 3 A spreadsheet file containing the cancer-essentiality results for genes and processes. (XLS 10752 kb)

Additional file 4 A spreadsheet file containing the drug prioritization results. (XLS 275 kb)

## Abbreviations

BRCA: breast carcinoma
CDK: cyclin dependent kinase
ER: estrogen receptor
ERBB2: erb-b2 receptor tyrosine kinase 2
FGFR: fibroblast growth factor receptor
GO: gene ontology
OVCA: serous ovarian adenocarcinoma
TCGA: The Cancer Genome Atlas
