# Supplementary material for: Data integration to prioritize drugs using genomics and curated data
Source: BioData Min. 2016 May 26;9:21. doi: 10.1186/s13040-016-0097-1 (PMC4881054; doi:10.1186/s13040-016-0097-1)
Supplement: Additional file 1 — A PDF-document containing the Supplementary Figures, Results and Methods. (PDF 273 kb) [file 13040_2016_97_MOESM1_ESM.pdf]

Supplementary documentation for "Data integration to prioritize drugs using genomics and curated data“

Riku Louhimo *, Marko Laakso *, Denis Belitskin, Juha Klefström, Rainer Lehtonen and Sampsa Hautaniemi

Faculty of Medicine, University of Helsinki, Finland
* Equal contribution
version: March 15, 2016

This documents contains a detailed description of the database and a formal description of the GOPredict algorithm as well as extended results. In addition, it contains a description of the analysis of in-house and curated data which the database contains as well as details of the preprocessing of query data sets used in the GOPredict case study.

Contents

1 Extended Results **2**
1.1 Supplementary Figures . . . 2
1.2 Gene cancer-essentiality (K-rank) . . . 2
1.3 Cancer-essential GO processes are closely connected to cancer hallmarks . 3
1.4 Cancer gene essentiality enables defining novel multivariate survival covariates . . . 4
1.5 GOPredict is robust to changes in study sets . . . 5

2 Extended Methods **5**
2.1 Preprocessing and analysis of in-house and curated data . . . 5
2.2 Preprocessing breast and ovarian cancer query data sets . . . 8
2.3 Survival analysis . . . 9

3 Database ranking and the GOPredict algorithm **9**
3.1 Overview of gene ranking in the database . . . 9
3.2 Overview of GOPredict . . . 10
3.3 Derivation of gene ranks . . . 10
3.4 Calculating the K-rank . . . 11
3.5 Essentiality scores . . . 11
3.6 Drug prioritization and activity matrix construction . . . 13

4 Table of data in each study and study set **14**

1 Extended Results

1.1 Supplementary Figures

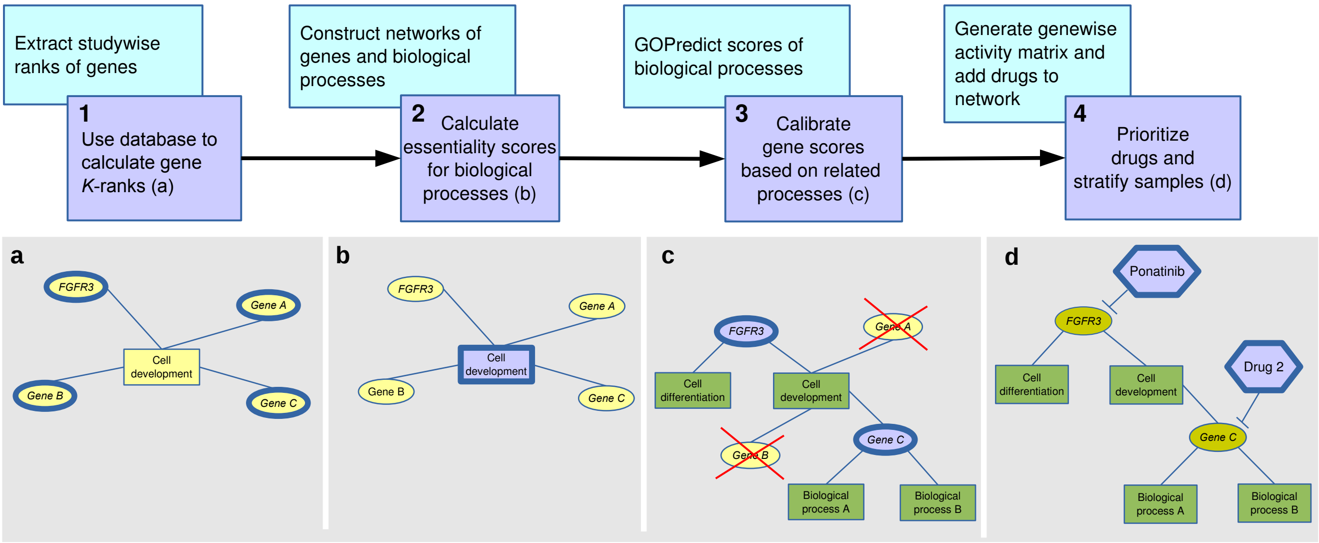

Figure 1: Details of GOPredict scoring. Circles are genes, boxes are GO processes and hexagons are drugs. Bold blue borders denote entity for which a score is being calculated. Green background denotes an entity whose scores have already been calculated. **(a)** Calculate K-ranks for genes. For example, the fibroblast growth factor receptor 3 (*FGFR3*) has a rank in two studies which are used to calculate its K-rank. **(b)** Calculate essentiality scores for GO processes using gene K-ranks. For example, 1,130 genes negatively and 939 positively regulate 'cell development' (GO:0048468) and have a K-rank in the database. Four genes are depicted for clarity. **(c)** Calibrate gene scores based on GO process essentiality scores: neighborhood of genes is expanded and genes without drugs are removed. For example, *FGFR3* unambiguously regulates 17 processes (9 positively, 8 negatively). Only two are depicted for clarity. **(d)** Prioritize drugs based on calibrated gene scores.

1.2 Gene cancer-essentiality (K-rank)

A byproduct of the knowledge-base design is that it allows defining hypothesis-driven selection of study sets. Here we used GOPredict to characterize the cancer-essentiality of genes in activating, inactivating and survival-associated study sets. A full list of studies in each study set is in Additional file 2.

We quantified cancer-essentiality for a gene with the K-rank separately for activating, inactivating and survival-associated study sets. Gene scores per study set are listed in Additional file 3. For the survival-associated data, we used studies containing results of univariate survival analyses. Different study sets contain different numbers of genes due to study specific inclusion criteria (Methods).

Out of 14,746 genes, 883 were significantly associated with activating changes (K-rank $P < 0.05$). Out of 17,560 genes, 1,245 were significantly associated with inactivating changes (K-rank $P < 0.05$). Seventy-five genes including *FGFR3* and *ERBB2* appear in both sets.

Out of 3,365 genes, 123 were significantly related to cancer survival in more than two studies (K-rank $P < 0.05$). Thirteen of these genes also appear in the inactivation and eight in the activation list. Of note, *EGFR* is significant in all three study sets: activating (2nd highest, $P = 7.0 \times 10^{-6}$), inactivating (11th, $P = 0.0003$) and survival-associated (7th, $P = 0.003$).

To summarize, the genes which GOPredict characterized to be cancer-essential include known cancer genes such as *EGFR* as well as genes not previously associated with cancer.

1.3 Cancer-essential GO processes are closely connected to cancer hallmarks

In addition to cancer-essentiality of genes, GOPredict quantifies the cancer-essentiality of GO processes. Out of 1,178 GO processes in our database, genes in the combined activating, inactivating and survival-associated set participated in 890 GO processes. Of the 7,474 regulatory relationships between genes and GO processes 4,343 were activating and 3,131 inhibitory.

Most genes regulated a small number of GO processes (mean 2.84, range 1–37). Interestingly, the 309 genes (12% of genes) which regulated more than five GO processes accounted for 43% of all activating interactions (Fisher's exact test $P = 0.0007$). Each GO process had 1 to 590 regulatory relationships (mean 10.3). Each GO process was regulated by a mean of 4.3 inhibitors and 6.0 activators which differed significantly (Mann-Whitney-U test $P = 2.1 \times 10^{-8}$).

Cancer-essentiality of a GO process was quantified separately for activation and inactivation. Thirty GO processes were both activated and inhibited in cancer (activation $P < 0.0001$ and inhibition $P < 0.0001$) and contain several GO processes highly connected to cancer hallmarks [1] (Additional file 3). Interestingly, the fibroblast growth

factor receptor signaling pathway (GO:0008543), which is known to play diverse roles in cancer in general [2] and female cancers in particular [3] was among the high scoring GO processes. In summary, the GO processes prioritized by our algorithm are known to be highly relevant for cancer as they capture known breast cancer as well as cancer-hallmark processes.

1.4 Cancer gene essentiality enables defining novel multivariate survival co-variates

GOPredict produces several by-product results when prioritizing drugs. For example, quantifying gene cancer-essentiality — an intermediary result of GOPredict — enables finding novel cancer gene candidates even when a gene is poorly characterized. As a proof of concept, we looked for genes that had received high scores through alterations in both ovarian and breast cancer study sets.

Three genes — *SLC25A32*, *PYCR1* and *OSR2* — were altered in one or more study sets of both cancers. For each gene separately, we built a multivariate Cox survival model with OVCA gene expression status (up/down/normal) of the gene, tumor grade, FIGO stage, and residual tumor size as co-variates. The overexpression of *SLC25A32* (ANOVA $P = 0.003$) and lack of residual tumor (ANOVA $P = 0.02$) were significant independent predictors of poor survival in TCGA OVCA (Table 1).

Table 1: **Analysis of Deviance table for the Cox regression model.**

**Co-variate**	**loglik**	**Chisq**	**Df**	$\mathbf{Pr}(>\|Chi\|)$	
SLC25A32 expression status	−1185.4	9.01	1	0.003	**
Grade	−1182.4	6.03	5	0.303	
FIGO Stage	−1174.3	16.1	9	0.07	.
Residual Tumor	−1169.2	10.3	3	0.016	*

Co-variate are the variates in the Cox model, loglik is the log-likelihood of co-variate, Chisq the chi-square statistic, Df the degress of freedom and $\mathbf{Pr}(> |Chi|)$ the P-value with this Chisq. In the P-value column, ** denotes $P < 0.01$, * $P < 0.05$ and . borderline significance.

1.5 GOPredict is robust to changes in study sets

We utilized GOPredict to analyze study sets which we defined to be activating, inactivating or survival-associated based on the type of studies in each set. To analyze the sensitivity of GOPredict to these choices, we added three TCGA methylation studies (BRCA, OVCA and COAD) to test the effect of changing study sets on the K-ranks and drug predictions in the BRCA *ERBB2* amplified query data set. The survival-associated K-ranks show good correlation (Spearman's $\rho = 0.97$). Analyzing the highest ranked 1,000 genes alone, K-ranks show very good correlation (Spearman's $\rho = 0.996$). Drug scores are also highly correlated (Spearman's $\rho = 0.86$) and the best scoring 10% of drugs (31 drugs) show an overlap of 90% (27/31) between the two analyses.

Furthermore, to test the effect of removing study sets on the results, we analyzed the BRCA *ERBB2* amplified query data set without the two Census studies. We then compared results with and without the Census data. Unadjusted K-ranks are highly correlated both for activating (Spearman's $\rho = 0.98$) and inactivating study sets (Spearman's $\rho = 0.99$). Furthermore, drug scores show high correlation (Spearman's $\rho = 0.95$). Thus, our results suggest that the Census studies are beneficial to include in the analysis but are not a major source of noise.

These analyses suggest that GOPredict scoring shows robustness to changes in study sets.

2 Extended Methods

All data were analyzed using the Anduril framework and Moksiskaan database integration tool [4, 5]. All annotations were based on the Ensembl GRCh37.

2.1 Preprocessing and analysis of in-house and curated data

First, we analyzed in-house four different cancers downloaded from The Cancer Genome Atlas (TCGA). TCGA provides a large set of tumor samples and related clinical data for various cancers [6]. We have previously analyzed gene-expression, copy-number and DNA methylation alterations in glioblastoma multiforme (GBM) [4] and serous ovarian carcinoma (OV) [7], and we also analyzed respective data in breast invasive carcinoma (BRCA) and colon adenocarcinoma (COAD). Furthermore, in all these data sets we analyzed the impact of gene expression, copy-number-alteration and DNA methylation on

patient survival. Statistical significance of survival was assessed in every instance using the log-rank test.

Second, we curated results from five different literature resources: the Catalogue of Somatic Mutation in Cancer (COSMIC) [8]; Tumorscape [9]; Cancer Gene Census [10]; Amplified and overexpressed genes in cancer [11]; and breast tumor brain metastases [12].

The high-confidence results from these analyses are stored in our database and these results are then used to calculate gene K-ranks and essentiality scores.

In-house data

We downloaded from the Cancer Genome Atlas two types of gene expression data (Agilent and AffyMetrix Exon array) for all four cancers. When both types of data were available for a cancer, only exon array data were used.

Expression data We preprocessed Agilent expression data (BRCA, COAD). First, probes matching either multiple or no genes were removed. Then, data were normalized to a mean of 0. Exon arrays (OV, GBM) were normalized and gene expression values quantified with the Multiple Exon Array Preprocessing algorithm (MEAP) [13]. After normalization, both platforms were further processed identically. For each gene, the genes was considered up- or downregulated in a sample if the gene's expression was further than three standard deviations from the median of control samples. We then grouped samples according to this up/downregulation data and analyzed the survival predictive power for each gene separately.

Copy-number data Copy-number data were also available from two platforms: AffyMetrix 6.0 SNP arrays (BRCA, COAD) and Agilent CGH (GBM, OV). When both platforms were available for a cancer, we used Agilent data. Copy-number data from AffyMetrix 6.0 SNP arrays were extracted with the R package crlmm [14]. Samples with signal-to-noise ratio of less than 5 were removed. Moreover, probes with confidence limit less than 0.9 were removed. Copy-number data from Agilent comparative genomic hybridization arrays were preprocessed as previously described in [4]. Data from both array platforms were segmented using circular binary segmentation [15]. Copy-number calls were made in two ways. For SNP arrays (BRCA, COAD), CNA were called when the copy-number was further than 0.3 from the diploid state. For Agilent CGH arrays (GBM, OV), copy-numbers were called as described in [7]. Copy-number calls per gene were used to group

samples for survival analysis so that deleted or amplified samples were compared against the non-deleted or non-amplified group of samples.

Methylation data We downloaded level 3 methylation data (beta values) and transformed them into M-values [16]. This transformation makes the methylation value distribution normal and enables us to use the t-test for methylation change significance analysis [16]. For each gene, if the genewise methylation difference between the median methylation of control samples and a tumor sample was more than 2 than the sample was grouped into hypo- or hypermethylated sample group. As in gene expression survival analysis, these groups were then used to determine methylation induced survival differences.

Database inclusion criteria All results were deposited genewise into our database. We filtered results to be deposited based on study-specific criteria. A gene's expression fold-change value was deposited into the database if the gene's $q \leq 0.001$ (t-test, Benjamini-Yakutieli multiple hypothesis correction). Similarly, survival analysis based on gene-expression data were deposited if the log-rank $p \leq 0.01$. For methylation data, methylation fold-changes were deposited if the $q \leq 0.001$ and methylation based log-rank survival $p \leq 0.01$. Copy-number alteration frequencies were deposited if the frequency exceeded 10%. Copy-number alteration based survival analysis results were saved if log-rank $p \leq 0.01$.

Curated data

The Catalogue of Somatic Mutation in Cancer (COSMIC) database is a collection of somatic aberrations of cancer genomes [8]. We obtained gene specific mutation frequencies from COSMIC with the Biomart interface. Tumorscape collects results of copy-number analyses of human cancers [9]. We included ten cancer types (breast, colorectal, glioma, hepatocellular, lung non-small cell carcinoma, lung squamous cell carcinoma, melanoma, ovarian, prostate, and renal) to our analyses. For each gene, we recorded whether it was amplified or deleted in a cancer type.

The Cancer Gene Census database provides information about causative mutations in cancer [10]. We used this database to identify genes, which are frequently activated (amplification, translocation) or inactivated (copy-number deletion, or missense-, nonsense- or splice-site-mutation) in cancer. Similarly, we obtained a list of genes for which amplification had been causally linked with the gene's overexpression and found a set of

77 frequently amplified and overexpressed genes in human cancers [11]. Lastly, we found a set of differentially expressed genes related to brain metastases in breast cancer [12]. We have included all 26 genes to this study.

COSMIC Mutation frequencies for each gene were saved if a mutation had been observed in at least 20 samples and the mutation ratio was at least 10%. Copy-number alterations from Tumorscape were saved if the genewise alteration frequency exceeded 10% and GISTIC $q \leq 0.25$.

2.2 Preprocessing breast and ovarian cancer query data sets

Gene expression data We downloaded gene expression microarrays from the Cancer Genome Atlas for 524 primary breast carcinoma tumors and 59 controls [17]. First, probes matching either multiple or no genes were removed. Then, data were normalized to a mean of 0. In addition, we downloaded exon expression microarrays for 491 ovarian serous adenocarcinoma tumors and 10 controls [18]. Exon arrays were normalized and gene expression values quantified with the Multiple Exon Array Preprocessing algorithm (MEAP) [13]. Each gene in each data set was assigned one of three states: upregulated, downregulated or normal. For both data sets, a gene was considered upregulated in a sample if that sample's expression was more than three standard deviations over the median of normal samples. Similarly, a gene was considered downregulated in a sample if that sample's expression was more than three standard deviations below the median of normal samples.

Genomic data Copy-number data from AffyMetrix 6.0 SNP arrays were extracted with the R package crlmm [14]. Samples with signal-to-noise ratio of less than 5 were removed. Moreover, probes with confidence limit less than 0.9 were removed. Copy-number data from Agilent comparative genomic hybridization (CGH) arrays were preprocessed as previously described [4]. Data from both array platforms were segmented using circular binary segmentation [15]. Copy-number calls were made in two ways. For SNP arrays (BRCA), CNA were called when the copy-number was further than 0.3 from the diploid state. For Agilent CGH arrays (OV), copy-numbers were called as previously described [7]. For each gene, copy-number was assigned to be either amplified, deleted, or unchanged. Mutation data were downloaded from TCGA and synonymous mutations were removed. All mutations were considered loss-of-function mutations. Mutation and copy-number were fused before building the activity matrix.

After preprocessing individual data levels, 497 BRCA and 390 OV samples with all three data types remained for the construction of activity matrices.

2.3 Survival analysis

To test the prognostic power of selected cancer-essential genes, we built a Cox proportional hazards model with the R package survival. The end-point was overall survival and events were defined as the patient vital status at last follow up. We developed a model for OVCA only for which there were 421 samples available with a total of 239 events.

The covariates included to the Cox model were the expression status of a gene (activated, inactivated or unchanged when compared to control), tumor grade, tumor FIGO stage, age at diagnosis (over 50yo versus at most 50yo), and the size of the residual tumor. All co-variates were categorical. Age was not included because it did not pass the proportional hazards assumption unlike the other covariates.

3 Database ranking and the GOPredict algorithm

The result database is an integrated database which contains relationships between bioentities such as genes, proteins, drugs, and biological processes [5]. The information has been integrated from multiple primary source databases including Ensembl, WikiPathways, Gene Ontology and PINA as well as drug target information from KEGG and DrugBank [5]. A complete and up-to-date list of databases included in the database is available from the website `http://csbi.ltdk.helsinki.fi/moksiskaan/`.

3.1 Overview of gene ranking in the database

The database contains analysis results in addition to relationships between bioentities. The results (studies) are stored as sets of bioentities (*e.g.,* genes) with a study specific value attached to each of them. For the GOPredict analysis, we extensively analyzed four TCGA data sets: ovarian [7], glioblastoma [4], breast and colorectal cancers. Briefly, data for gene copy-number (SNP and Array CGH), transcriptional expression, DNA methylation and clinical data are analyzed and the high-confidence results are stored in the database. For example, *ERBB2* is overexpressed in our TCGA breast cancer transcriptomic analysis and subsequently stored in the database as an overexpressed bioentity (gene) in a specific study (TCGA breast cancer transcriptomic analysis). Similar ranks

have been derived for all genes from other analyses. The per-gene database ranks are used to calculate for each gene a cancer essentiality score called the K-rank (Figure 1).

3.2 Overview of GOPredict

Details of GOPredict are shown in Figure 1. Study specific gene K-ranks are calculated for three subsets of studies in the database (Table 2). These gene specific K-ranks are then mapped to Gene Ontology processes, and recalibrated K-ranks or cancer essentiality scores for genes are calculated based on the process scores. Finally, the recalibrated K-ranks are used to determine drug prioritization scores. Drugs targeting genes, which are deleted, do not influence the score calculation.

3.3 Derivation of gene ranks

Let C denote the set of all studies stored in the Moksiskaan database (2) and G denote all genes in the database (note that this set contains all primary assembly Ensembl v.70 genes without patches). Every study in the database is a set of result values (*e.g.,* copy-number alteration frequencies, gene expression fold-change values, somatic mutation frequency according to COSMIC) for genes. Let study $m \in C$ and gene $g \in G$, and let $\mathbf{V} \in \mathbb{R}^{G \times C}$ contain the scores of all genes in all studies. Moreover, let $v_{g,m} \in \mathbf{V}$ be the score of gene g in study m.

Since scores from different studies originate from different measurements with different dynamic ranges, the most robust way of combining them is by ranking them according to their relative score in each study. We also have to take ties into account because gene scores can contain duplicated values.

The function $o_+ : G \times C \to [0, |G| - 1]$ provides the number of values in $\mathbf{V}$ which are greater than the given (x,y):

$$o_+(x, y) = |\{\forall w \in G \,|\, \mathbf{V}_{w,y} > v_{x,y}\}|,\ x \in G, y \in C$$

Similarly, the function $o_= : G \times C \to [0, |G|]$ provides the number of values in $\mathbf{V}$ equal to the given (x,y):

$$o_=(x, y) = |\{\forall w \in G \,|\, \mathbf{V}_{w,y} = v_{x,y}\}|,\ x \in G, y \in C$$

Lastly, let $L_m = \{\forall w \in G \,|\, \mathbf{V}_{w,m} > 0\}$. The rank values $\mathbf{W} \in \mathbb{R}^{G \times C}$ are calculated for each $g \in G$ as follows:

$$\mathbf{W_{g,m}} = \begin{cases} 1 - \frac{o_{+}(g,m)+\frac{o_{=}(g,m)-1}{2}}{|L_m|+1}, & v_{g,m} > 0 \\ 0, & \text{otherwise} \end{cases} \tag{1}$$

where $\frac{o_{=}(g,m)-1}{2}$ balances the effect of duplicate scores by averaging the number of scores equal to $v_{g,m}$ (not including g itself, hence subtracting one).

Normalized rank values ($\mathbf{N} \in \mathbb{R}^{G \times C}$) for each m are produced by scaling the sum of each column $\mathbf{W}_{:,m}$:

$$\mathbf{N}_{g,m} = \frac{\mathbf{W}_{g,m}}{\sum \mathbf{W}_{:,m}} \tag{2}$$

3.4 Calculating the K-rank

Recall that $m \in C$ and $g \in G$, and let $\mathbf{k} \in \mathbb{R}^{G \times 1}$ be a matrix of studywise importance scores of genes. Now $\mathbf{k}$ is derived from the normalized ranks of a particular set of studies $C' \subset C$:

$$\mathbf{k}_g = \frac{\sum^{C'} \mathbf{N}_{g,C'}}{|C'|} \tag{3}$$

Similarly to $\mathbf{k}$, let $\mathbf{m} \in \mathbb{R}^{G \times 1}$, $\mathbf{o} \in \mathbb{R}^{G \times 1}$ and $\mathbf{r} \in \mathbb{R}^{G \times 1}$ be matrices derived from study subsets of C: the activated, inactivated and survival-associated subsets (as listed in Table 2). These score matrices represent three separate scores: up-regulation score $\mathbf{m}$ indicating activation of a gene in cancer, down-regulation score $\mathbf{o}$ representing deactivation of a gene in cancer, and an unsigned survival-associated score $\mathbf{r}$.

3.5 Essentiality scores

We start by defining the **permutation test function** which will be used to assess statistical significance of the scores. Let $perm(\mathbf{a}, \mathbf{y}, \Gamma) : \mathbb{R} \rightarrow \mathbb{R}^{j \times 1}$ be the permutation test function, where j is the number of tests; $\mathbf{a} \in \mathbb{R}^{j \times 1}$ is the vector of observed values of the test statistic; $\mathbf{y} \in \mathbb{N}^{j \times 1}$ is a vector of sample sizes which is randomly sampled for each $a_j \in \mathbf{a}$; and Γ is the sample space from which $y_j \in \mathbf{y}$ values are drawn in each permutation for each j. Moreover, let 5,000 be the number of permutations.

Calculating Gene Ontology process essentiality scores

Let $\mathbf{u} = \mathbf{m} + \mathbf{r},\ \mathbf{u} \in \mathbb{R}^{G\times 1}$ be the upregulation scores for all genes $g \in G$ and $\mathbf{d} = \mathbf{o} + \mathbf{r},\ \mathbf{d} \in \mathbb{R}^{G\times 1}$ the downregulation scores. In addition, let B be the set of biological process in Gene Ontology (GO) and let $b \in B$ be a biological process (**Fig 1a**). Moreover, let $\mathbf{R}^{+} \in \{0,1\}^{G\times B}$ and $\mathbf{R}^{-} \in \{0,1\}^{G\times B}$ be binary matrices such that

$$\mathbf{R}^{+}_{g,b} = \begin{cases} 1, & \text{if } g \text{ positively regulates } b \\ 0, & \end{cases}$$

and

$$\mathbf{R}^{-}_{g,b} = \begin{cases} 1, & \text{if } g \text{ negatively regulates } b \\ 0. & \end{cases}$$

Essentiality scores $\mathbf{s2}^{+} \in \mathbb{R}^{B\times 1}$ and $\mathbf{s2}^{-} \in \mathbb{R}^{B\times 1}$ for GO processes (**Fig 1b**) are row sums of the product of a score vector ($\mathbf{u}$ or $\mathbf{d}$) and its regulation matrix ($\mathbf{R}^{+}$ or $\mathbf{R}^{-}$):

$$\mathbf{s2}^{+}_{b} = \sum(\mathbf{R}^{+}_{:,b}\mathbf{u}) \tag{4}$$

and

$$\mathbf{s2}^{-}_{b} = \sum(\mathbf{R}^{-}_{:,b}\mathbf{d}) \tag{5}$$

Note that $\sum \mathbf{R}^{+}_{:,b} \in \mathbb{N}^{B\times 1}$ is the vector of all positively regulating edge counts of GO processes b and similarly $\sum \mathbf{R}^{-}_{:,b} \in \mathbb{N}^{B\times 1}$ for negatively regulating. P-value vectors $\mathbf{p2}^{+} \in \mathbb{R}^{B\times 1}$ and $\mathbf{p2}^{-} \in \mathbb{R}^{B\times 1}$ for $\mathbf{s2}^{+}$ and $\mathbf{s2}^{-}$ are calculated as

$$\mathbf{p2}^{+}_{b} = perm(\mathbf{s2}^{+}, \sum \mathbf{R}^{+}_{:,b}, \mathbf{u}) \tag{6}$$

and

$$\mathbf{p2}^{-}_{b} = perm(\mathbf{s2}^{-}, \sum \mathbf{R}^{-}_{:,b}, \mathbf{d}) \tag{7}$$

Recalibrating the K-rank

Let B_1 and B_2 be sets of GO processes such that $B_1 = \{\forall q \in B_1 \,|\, \mathbf{p2}^{+}_{q} \leq \mathbf{p2}^{-}_{q}\}$ and $B_2 = \{\forall a \in B_2 \,|\, \mathbf{p2}^{-}_{a} \leq \mathbf{p2}^{+}_{a}\}$. Recalibrated K-ranks or gene essentiality scores (**Fig 1c**) $\mathbf{s3}^{+} \in \mathbb{R}^{G\times 1}$ and $\mathbf{s3}^{-} \in \mathbb{R}^{G\times 1}$ are defined as

$$\mathbf{s3}_g^+ = \frac{|B_1| + |B_2|}{\sum^{B_1}\left(\frac{1}{\mathbf{R}_{g,B_1}^+ \mathbf{p2}^+}\right) + \sum^{B_2}\left(\frac{1}{\mathbf{R}_{g,B_2}^- \mathbf{p2}^-}\right)} \tag{8}$$

and

$$\mathbf{s3}_g^- = \frac{|B_2| + |B_1|}{\sum^{B_2}\left(\frac{1}{\mathbf{R}_{g,B_2}^+ \mathbf{p2}^-}\right) + \sum^{B_1}\left(\frac{1}{\mathbf{R}_{g,B_1}^- \mathbf{p2}^+}\right)} \tag{9}$$

Thus, essentiality scores $s3_g^+$ and $s3_g^-$ for a gene g are the harmonic means of all P-values of GO processes it regulates. P-values for all $\mathbf{s3}^+$ and $\mathbf{s3}^-$ are calculated by a permutation test and then transformed to logarithmic scale. First, let $\mathbf{q} = [\begin{smallmatrix}\mathbf{u}\\\mathbf{d}\end{smallmatrix}]$ and $\mathbf{p3}^+ \in \mathbb{R}^{G\times 1}$ and $\mathbf{p3}^- \in \mathbb{R}^{G\times 1}$. Now

$$\mathbf{p3}_g^+ = -\log_2 \left[perm\left(\mathbf{s3}^+, \sum(\mathbf{R}_{g,:}^+ + \mathbf{R}_{g,:}^-), \mathbf{q}\right)\right] \tag{10}$$

and

$$\mathbf{p3}_g^- = -\log_2 \left[perm\left(\mathbf{s3}^-, \sum(\mathbf{R}_{g,:}^- + \mathbf{R}_{g,:}^+), \mathbf{q}\right)\right] \tag{11}$$

3.6 Drug prioritization and activity matrix construction

In the final step, we add drugs and sample specific measurements to the network. Measurements of biological alterations of each sample are represented as graphs of genes and their activities. Let S be the set of samples. The activity status matrix $\mathbf{S} \in \{-2, -1, 0, 1\}^{G\times S}$ of sample genes $g \in G$ and samples $s \in S$ is inferred from the matrix of transcriptional activities $\mathbf{E} \in \{-1, 0, 1\}^{G\times S}$ integrated with DNA mutations and copy-number alterations $\mathbf{A} \in \{-1, 0, 1\}^{G\times S}$. Furthermore, let $e_{g,s} \in \mathbf{E}$ and $\alpha_{g,s} \in \mathbf{A}$:

$$\mathbf{S}_{g,s} = \begin{cases} 1, & e_{g,s} = 1 \vee (e_{g,s} \notin \{-1, 0, 1\} \wedge \alpha_{g,s} = 1) \\ 0, & e_{g,s} = 0 \\ -1, & e_{g,s} = -1 \\ -2, & \alpha_{g,s} < 0 \\ \text{NA} & \text{otherwise.} \end{cases} \tag{12}$$

Let D be the set of all drugs in Moksiskaan and $d \in D$. Drugs are added if they regulate genes in G. Let $\mathbf{D} \in \{-1, 0, 1\}^{D\times G}$ be a matrix of effects of drugs on the genes $g \in G$ such that

$$\mathbf{D}_{d,g} = \begin{cases} 1, & \text{if } d \text{ activates } g \\ -1, & \text{if } d \text{ inhibits } g \\ 0. & \end{cases}$$

For each gene, we know the sets of samples that are either up- or downregulated in the sample set. Accordingly, let $S_g^+ = \{\forall x \in S \,|\, \mathbf{S}_{g,x} > 0\} \subseteq S$ for upregulation and $S_g^- = \{\forall y \in S \,|\, \mathbf{S}_{g,y} < 0\} \subseteq S$ for downregulation. Observe that $\forall g : S_g^+ \cap S_g^- = \emptyset$. Furthermore, let $G_d^+ = \{\forall x \in G \,|\, \mathbf{D}_{d,x} = 1\}$ be the set of genes with an activating drug and $G_d^- = \{\forall x \in G \,|\, \mathbf{D}_{d,x} = -1\}$ be the set of genes with an inactivating drug. The drug scores $\mathbf{s4}^+ \in \mathbb{R}^{D\times 1}$ and $\mathbf{s4}^- \in \mathbb{R}^{D\times 1}$ (**Fig 1d**) for each drug $d \in D$ over the whole sample set S are

$$\mathbf{s4}_d^+ = \sum_{x\in S_g^+} \sum_{g\in G_d^+} \frac{\mathbf{S}_{g,x}\mathbf{D}_{d,g}\mathbf{p3}_g^+}{|S_g^+|} \tag{13}$$

and

$$\mathbf{s4}_d^- = \sum_{x\in S_g^-} \sum_{g\in G_d^-} \frac{\mathbf{S}_{g,x}\mathbf{D}_{d,g}\mathbf{p3}_g^-}{|S_g^-|} \tag{14}$$

Sorted lists of $\mathbf{s4}^+$ and $\mathbf{s4}^-$ scores yield the prioritized drugs with the largest effect in the input cohort.

4 Table of data in each study and study set

Table 2: Descriptions of the cancer data sets stored in Moksiskaan. As detailed in the *study sets* column, these studies are used for the matrices **m** (activating study set), **o** (inactivating), and **r** (survival-associated). The numbers of results reported and their units are reported in *number of genes* and *score type* columns, respectively.

name	description	number of genes	score type	study sets
cancerGeneCensusAct	Frequent activating genetic alterations in cancer [10]	321	Descending rank	**m**
cancerGeneCensusIna	Frequent inactivating genetic alterations in cancer [10]	152	Descending rank	**o**
cosmicMetastasis	Consists of frequent somatic mutations in metastasis tumours as reported in COSMIC database. [8, 19]	2308	Proportion	**o**
cosmicPrimary	Consists of frequent somatic mutations in primary tumours as reported in COSMIC database. [8, 19]	10335	Proportion	**o**
cosmicRecurrent	Consists of frequent somatic mutations in recurrent tumours as reported in COSMIC database. [8, 19]	581	Proportion	**o**
fileAmpOver	Amplified and overexpressed genes in human cancer. [11]	77	Ascending rank	**m**
fileBC2brain	Set of differentially expressed genes related to brain metastases of the breast cancer tumor. [12]	26	Fold change	**m**

Continued on next page...

name	description	number of genes	score type	study sets
tcgaBreastCGHa	CGH gains in TCGA Breast samples	9422	Proportion	**m**
tcgaBreastCGHd	CGH losses in TCGA Breast samples	2225	Proportion	**o**
tcgaBreastCGHSurv	Survival associated copy-number aberrations in TCGA Breast samples	2123	Proportion	**r**
tcgaBreastGE	Differentially expressed genes in TCGA Breast samples	9109	Fold change	$\mathbf{m} > 1$, $\mathbf{o} < 1$
tcgaBreastGESurv	Genes with survival associated expressions in TCGA Breast samples	268	Probability	**r**
tcgaBreastMethylSurv	Genes with survival assodicated methylation differences in TCGA Breast samples	128	Probability	**r**
tcgaColonCGHa	CGH gains in TCGA Colon samples	1194	Proportion	**m**
tcgaColonCGHd	CGH losses in TCGA Colon samples	11	Proportion	**o**
tcgaColonGE	Differentially expressed genes in TCGA Colon samples	8688	Fold change	$\mathbf{m} > 1$, $\mathbf{o} < 1$
tcgaColonGESurv	Genes with survival associated expressions in TCGA Colon samples	88	Probability	**r**
tcgaColonMethylSurv	Genes with survival assodicated methylation differences in TCGA Colon samples	372	Probability	**r**
tcgaGliomaCGHa	CGH gains in TCGA Glioma samples [4]	25	Proportion	**m**
tcgaGliomaCGHd	CGH losses in TCGA Glioma samples [4]	465	Proportion	**o**
tcgaGliomaCGHSurv	Survival associated copy-number aberrations in TCGA Glioma samples [4]	7	Proportion	**r**
tcgaGliomaGE	Differentially expressed genes in TCGA Glioma samples [4]	5847	Fold change	$\mathbf{m} > 1$, $\mathbf{o} < 1$
tcgaGliomaGESurv	Genes with survival associated expressions in TCGA Glioma samples [4]	167	Probability	**r**
tcgaGliomaMethylSurv	Genes with survival assodicated methylation differences in TCGA Glioma samples [4]	330	Probability	**r**
tcgaOvarianCGHa	CGH gains in TCGA Ovarian samples [7]	546	Proportion	**m**
tcgaOvarianCGHd	CGH losses in TCGA Ovarian samples [7]	173	Proportion	**o**
tcgaOvarianCGHSurv	Survival associated copy-number aberrations in TCGA Ovarian samples [7]	127	Proportion	**r**
tcgaOvarianGE	Differentially expressed genes in TCGA Ovarian samples [7]	505	Fold change	$\mathbf{m} > 1$, $\mathbf{o} < 1$
tcgaOvarianGESurv	Genes with survival associated expressions in TCGA Ovarian samples [7]	31	Probability	**r**
tcgaOvarianMethylSurv	Genes with survival assodicated methylation differences in TCGA Ovarian samples [7]	144	Probability	**r**
tscapeBCa	Frequent (minFreq=0.1, maxQ=0.25) chromosomal amplifications in Breast tumours [9]	688	Proportion	**m**
tscapeBCd	Frequent (minFreq=0.1, maxQ=0.25) chromosomal deletions in Breast tumours [9]	1616	Proportion	**o**
tscapeCRCa	Frequent (minFreq=0.1, maxQ=0.25) chromosomal amplifications in Colorect tumours [9]	213	Proportion	**m**
tscapeCRCd	Frequent (minFreq=0.1, maxQ=0.25) chromosomal deletions in Colorectal tumours [9]	856	Proportion	**o**
tscapeGliomaa	Frequent (minFreq=0.1, maxQ=0.25) chromosomal amplifications in Glioma tumours [9]	24	Proportion	**m**
tscapeGliomad	Frequent (minFreq=0.1, maxQ=0.25) chromosomal deletions in Glioma tumours [9]	87	Proportion	**o**
tscapeHCCa	Frequent (minFreq=0.1, maxQ=0.25) chromosomal amplifications in Hepatocellular tumours [9]	323	Proportion	**m**
tscapeHCCd	Frequent (minFreq=0.1, maxQ=0.25) chromosomal deletions in Hepatocellular tumours [9]	606	Proportion	**o**
tscapeMelanomaa	Frequent (minFreq=0.1, maxQ=0.25) chromosomal amplifications in Melanon tumours [9]	326	Proportion	**m**
tscapeMelanomad	Frequent (minFreq=0.1, maxQ=0.25) chromosomal deletions in Melanoma tumours [9]	374	Proportion	**o**
tscapeNSCLCa	Frequent (minFreq=0.1, maxQ=0.25) chromosomal amplifications in Lung NS tumours [9]	1068	Proportion	**m**
tscapeNSCLCd	Frequent (minFreq=0.1, maxQ=0.25) chromosomal deletions in Lung NSC tumours [9]	1792	Proportion	**o**
tscapeOvariana	Frequent (minFreq=0.1, maxQ=0.25) chromosomal amplifications in Ovarian tumours [9]	908	Proportion	**m**
tscapeOvariand	Frequent (minFreq=0.1, maxQ=0.25) chromosomal deletions in Ovarian tumours [9]	1470	Proportion	**o**
tscapeProstatea	Frequent (minFreq=0.1, maxQ=0.25) chromosomal amplifications in Prostate tumours [9]	70	Proportion	**m**
tscapeProstated	Frequent (minFreq=0.1, maxQ=0.25) chromosomal deletions in Prostate tumours [9]	706	Proportion	**o**
tscapeRCCa	Frequent (minFreq=0.1, maxQ=0.25) chromosomal amplifications in Renal tumours [9]	163	Proportion	**m**
tscapeRCCd	Frequent (minFreq=0.1, maxQ=0.25) chromosomal deletions in Renal tumours [9]	713	Proportion	**o**
tscapeSCLCa	Frequent (minFreq=0.1, maxQ=0.25) chromosomal amplifications in Lung SC tumours [9]	196	Proportion	**m**

Continued on next page...

name	description	number of genes	score type	study sets
tscapeSCLCd	Frequent (minFreq=0.1, maxQ=0.25) chromosomal deletions in Lung SC tumours [9]	309	Proportion	**o**

Additional Files

Additional file 1

This file.

Additional file 2

A spreadsheet file containing the list of studies.

Additional file 3

A spreadsheet file containing the cancer-essentiality results for genes and processes.

Additional file 4

A spreadsheet file containing the drug prioritization results.

Additional file 5

A ZIP-archive containing the analysis code.

References

[1] Hanahan, D., Weinberg, R. Hallmarks of cancer: the next generation. *Cell* **144**, 646–674 (2011).

[2] Turner, N., Grose, R. Fibroblast growth factor signalling: from development to cancer. *Nat Rev Cancer* **10**, 116–129 (2010).

[3] Fearon, A. E., Gould, C. R., Grose, R. P. FGFR signalling in women's cancers. *Int J Biochem Cell Biol* **45**, 2832–2842 (2013).

[4] Ovaska, K., Laakso, M., *et al.* Large-scale data integration framework provides a comprehensive view on glioblastoma multiforme. *Genome Med.* **2**, 65 (2010).

[5] Laakso, M., Hautaniemi, S. Integrative platform to translate gene sets to networks. *Bioinformatics* **26**, 1802–1803 (2010).

[6] The Cancer Genome Atlas Research Network. Comprehensive genomic characterization defines human glioblastoma genes and core pathways. *Nature* **455**, 1061–1068 (2008).

[7] Louhimo, R., Hautaniemi, S. CNAmet: an R package for integration of copy number, expression and methylation data. *Bioinformatics* **27**, 887–888 (2011).

[8] Forbes, S., Bindal, N., Bamford, S., Cole, C., Kok, C., *et al.* COSMIC: mining complete cancer genomes in the Catalogue of Somatic Mutations in Cancer. *Nucleic Acids Research* **39**, D945 (2011).

[9] Beroukhim, R., Mermel, C. H., *et al.* The landscape of somatic copy-number alteration across human cancers. *Nature* **463**, 899–905 (2010).

[10] Futreal, P. A., Coin, L., Marshall, M., Down, T., Hubbard, T., *et al.* A census of human cancer genes. *Nature Reviews Cancer* **4**, 177–183 (2004).

[11] Santarius, T., Shipley, J., Brewer, D., Stratton, M., Cooper, C. A census of amplified and overexpressed human cancer genes. *Nature Reviews Cancer* **10**, 59–64 (2010).

[12] Bos, P., Xiang, H., Nadal, C., Shu, W., Gomis, R., *et al.* Genes that mediate breast cancer metastasis to the brain. *Nature* **459**, 1005 (2009).

[13] Chen, P., Lepikhova, T., *et al.* Comprehensive exon array data processing method for quantitative analysis of alternative spliced variants. *Nucleic Acids Res.* **39**, e123 (2011).

[14] Carvalho, B., Bengtsson, H., Speed, T. P., Irizarry, R. A. Exploration, normalization, and genotype calls of high-density oligonucleotide SNP array data. *Biostatistics* **8**, 485–499 (2007).

[15] Olshen, A. B., Venkatraman, E. S., *et al.* Circular binary segmentation for the analysis of array–based DNA copy number data. *Biostatistics* **5**, 557–572 (2004).

[16] Du, P., Zhang, X., Huang, C., Jafari, N., Kibbe, W., *et al.* Comparison of Beta-value and M-value methods for quantifying methylation levels by microarray analysis. *BMC Bioinformatics* **11**, 587 (2010).

[17] The Cancer Genome Atlas Network. Comprehensive molecular portraits of human breast tumours. *Nature* **490**, 61–70 (2012).

[18] The Cancer Genome Atlas Network. Integrated genomic analyses of ovarian carcinoma. *Nature* **474**, 609–615 (2011).

[19] Forbes, S., Bhamra, G., Bamford, S., Dawson, E., Kok, C., *et al.* The catalogue of somatic mutations in cancer (COSMIC). *Curr Protoc Hum Genet* **Chapter 10** (2008).
